# Nullomers and High Order Nullomers in Genomic Sequences

**DOI:** 10.1371/journal.pone.0164540

**Published:** 2016-12-01

**Authors:** Davide Vergni, Daniele Santoni

**Affiliations:** 1 Istituto per le Applicazioni del Calcolo “Mauro Picone” - CNR, Via dei Taurini 19, 00185, Rome, Italy; 2 Istituto di Analisi dei Sistemi ed Informatica “Antonio Ruberti” - CNR, Via dei Taurini 19, 00185, Rome, Italy; University of Münster, GERMANY

## Abstract

A nullomer is an oligomer that does not occur as a subsequence in a given DNA sequence, i.e. it is an absent word of that sequence. The importance of nullomers in several applications, from drug discovery to forensic practice, is now debated in the literature. Here, we investigated the nature of nullomers, whether their absence in genomes has just a statistical explanation or it is a peculiar feature of genomic sequences. We introduced an extension of the notion of nullomer, namely high order nullomers, which are nullomers whose mutated sequences are still nullomers. We studied different aspects of them: comparison with nullomers of random sequences, CpG distribution and mean helical rise. In agreement with previous results we found that the number of nullomers in the human genome is much larger than expected by chance. Nevertheless antithetical results were found when considering a random DNA sequence preserving dinucleotide frequencies. The analysis of CpG frequencies in nullomers and high order nullomers revealed, as expected, a high CpG content but it also highlighted a strong dependence of CpG frequencies on the dinucleotide position, suggesting that nullomers have their own peculiar structure and are not simply sequences whose CpG frequency is biased. Furthermore, phylogenetic trees were built on eleven species based on both the similarities between the dinucleotide frequencies and the number of nullomers two species share, showing that nullomers are fairly conserved among close species. Finally the study of mean helical rise of nullomers sequences revealed significantly high mean rise values, reinforcing the hypothesis that those sequences have some peculiar structural features. The obtained results show that nullomers are the consequence of the peculiar structure of DNA (also including biased CpG frequency and CpGs islands), so that the hypermutability model, also taking into account CpG islands, seems to be not sufficient to explain nullomer phenomenon. Finally, high order nullomers could emphasize those features that already make simple nullomers useful in several applications.

## Introduction

In the post genomic era a growing number of genomes has been completely sequenced and made available. In 1995 for the first time the genome of a living organism, Haemophilus influenzae, a Gram-negative anaerobic bacterium, was completely sequenced [1]. Advanced technologies, based on “shotgun” sequencing, and the availability of new massive computational resources led to a multitude of whole genomic sequences. New ambitious projects were designed, leading to the completion of the genome of bacterium Escherichia coli K-12 and the yeast Saccharomyces cerevisiae; finally the Human Genome Project was completed in 2005. As a great number of genomes was made available, scientists started to study and compare genome features in terms of similarity, complexity, information content and statistical properties. The first whole-genome studies provided insights on genome composition in terms of subsequences or k-mers [2, 3]. In recent years the term “nullomer” was used for the first time to indicate an absent word of a given genomic sequence or of a collection of sequences [4]; further investigations were conducted in [5, 6]. The set of nullomers of a given length associated to a genome is the collection of sequences of that length that do not occur in any chromosome of the genome as substrings. A refinement of the concept of nullomers was due to [7] where minimal absent words were introduced.

Why are nullomers studied in genomics and what kind of insights can they provide? The first issue concerns understanding why nullomers exist in the genomes: is it just a statistical matter or is it due to peculiar features of genomic sequences? The work of [5] suggests that the origin of nullomers is the hypermutability of CpG dinucleotides [8], while other works [9–11] show a more complex scenario.

In this work we investigated the structure of nullomers both theoretically and numerically. First of all we extended the notion of nullomer, introducing high order nullomers, i.e., nullomers whose mutated sequences are still nullomers. For instance, a k-mer that is not present in a DNA-sequence together with all its possible two letters mutations is called a second order nullomer. We compared the sets of simple (i.e., zero order nullomers; for the sake of simplicity and when there is no ambiguity we refer to zero order nullomers as “nullomers”) and high order nullomers of the human genome, with those expected in random sequences, preserving nucleotide and dinucleotide frequencies. Then we investigated the nature of simple and high order nullomers, studying their peculiar patterns in terms of both dinucleotide composition and physical chemical properties. Finally we built phylogenetic trees using simple and high order nullomers; the consistence of those trees with respect to classical phylogeny revealed that nullomers are well conserved among close species.

## Materials and Methods

### Nullomers and high order nullomers

Here we mathematically define nullomers and high order nullomers. We use the term *sequence* to indicate a long sequence (as a genome), and the term *word* to indicate a small sequence (as a k-mer).

Let *A* = {*a*_1_, *a*_2_, …, *a*_*m*_} be a finite set that we call alphabet (in our case *A* = {*a*, *c*, *g*, *t*}). Let *A*^*d*^ = {*x*_1_ *x*_2_…*x*_*d*_|*x*_*i*_ ∈ *A*, *i* = 1…*d*} be the set of all the possible sequences of length *d* on the alphabet *A*, and $A^{*}=\cup_{i=1}^{\infty }A^{i}\cup \;\epsilon$ be the set of all possible sequences on *A* of any length, including the empty word *ϵ*.

**Definition** Given a sequence, *w* ∈ *A*^*n*^ = *x*_1_ *x*_2_…*x*_*n*_, we define the set of all the words of length *d* (*d* ≪ *n*) that do not occur in *w* as

$$\begin{array}{c} H_{0}^{d}\left(w\right)=\left\{u\in A^{d}\;|\;∄\;s,t\in A^{*}\;\;\text{so}\;\text{that}\;\;w=sut\right\}. \end{array}$$ (1)

Each element of the above defined set is a nullomer of the sequence *w*.

In order to define high-order nullomers it is necessary to introduce the concept of mutation of a sequence. Given a word *u* = *y*_1_ *y*_2_…*y*_*d*_ ∈ *A*^*d*^, we define the first order set of mutations of *u* as the set of words that differ from *u* at most for one letter:

$$\begin{array}{ccc} N_{1}\left(u\right) & = & \{y_{1}...y_{j-1}\;b\;y_{j+1}...y_{d}\;\forall \;\;b\in A\;\;\text{and}\;\;j\in \left[1...d\right]\\ & & |\;\;y_{1}y_{2}...y_{d}=u\}\;. \end{array}$$ (2)

Then we define the second order set of mutations of *u* as the set of words that differ from *u* at most for two letters:

$$\begin{array}{ccc} N_{2}\left(u\right) & = & \{y_{1}...y_{j-1}\;b\;y_{j+1}...y_{d}\;\forall \;\;b\in A\;\;\text{and}\;\;j\in \left[1...d\right]\\ & & |\;\;y_{1}y_{2}...y_{d}\in N_{1}\left(u\right)\}\;. \end{array}$$ (3)

It is easy to generalize such a construction to define the set of mutations of order *s* of *u*:

$$\begin{array}{ccc} N_{s}\left(u\right) & = & \{y_{1}...y_{j-1}\;b\;y_{j+1}...y_{d}\;\forall \;\;b\in A\;\;\text{and}\;\;j\in \left[1...d\right]\\ & & |\;\;y_{1}y_{2}...y_{d}\in N_{s-1}\left(u\right)\}\;. \end{array}$$ (4)

In such a way we obtain a collection of sets *N*_*s*_(*u*) with *s* = 1, 2, 3, … such that:

$$\begin{array}{c} \left\{u\right\}\subseteq N_{1}\left(u\right)\subseteq N_{2}\left(u\right)\subseteq ...\subseteq N_{s}\left(u\right)\subseteq ... \end{array}$$ (5)

**Definition** A word *u* is a nullomer of order *s* of a given sequence *w* if all the elements in *N*_*s*_(*u*) are nullomers of *w*.

The set of all nullomers of order *s* for a given sequence *w* is defined as

$$\begin{array}{c} H_{s}^{d}\left(w\right)=\left\{u\in A^{d}\;|\;\forall v\in N_{s}\left(u\right)\Rightarrow v\in H_{0}^{d}\left(w\right)\right\} \end{array}$$ (6)

We obtain a collection of sets $H_{s}^{d}\left(w\right)$ such that:

$$\begin{array}{c} H_{0}^{d}\left(w\right)⊇H_{1}^{d}\left(w\right)⊇H_{2}^{d}\left(u\right)⊇...⊇H_{s}^{d}\left(w\right)⊇... \end{array}$$ (7)

Trivially the higher the order the smaller the number of nullomers will be.

The classes of sets defined above can be easily extended in the case of a collection of sequences instead of a single sequence. Let *W* = {*w*_1_, *w*_2_, …, *w*_*n*_} a collection of sequences such that *w*_*i*_ ∈ A*, we define the set $H_{s}^{d}\left(W\right)$ as the intersection of all $H_{s}^{d}\left(w_{i}\right)$:

$$\begin{array}{c} H_{s}^{d}\left(W\right)=\overset{n}{\underset{i=1}{⋂}}H_{s}^{d}\left(w_{i}\right) \end{array}$$ (8)

It has been necessary to introduce such a generalization since genomes consist in a collection of sequences.

As an example, we consider simple nullomers and first order nullomers of the short sequence *w* = *acgttatgacaggcctgtcataacgt. It is very simple to see that all sequences of size 2 are present, while there are many absent sequences of size 3, e.g. aaa, aag, aat, …. Regarding first order nullomers, the minimal size at which they appear is 4. Just to pick an example, the nullomer cccc is a first order nullomer since each sequence obtained from it by mutating one nucleotide is a nullomer, while the nullomer aaaa is not a first order nullomer since its mutated sequence ataa is present as a subsequence of w*.

### Statistics of nullomers and high order nullomers in random sequences

The search for absent words in random and non-random sequences is a well-posed mathematical problem; nevertheless it has not received much attention in the literature (the main contribution in this field comes from [12–14]). Here we propose a direct and intuitive approach able to provide a good approximation of the probability for a word to be absent in a long and random symbolic sequence. Following [6] we used the Poisson probability distribution as a first approximation; however, differently from the above cited paper, we also considered both the periodicity of words, which is a fundamental property to be taken into account (see [12]), and different random processes underlying the generation of the symbolic sequence. Finally we generalized the computation to consider high order nullomers.

A word *u* ∈ *A*^*d*^, *u* = *y*_1_ *y*_2_…*y*_*d*_, is periodic of period *t* if a shift of *t* positions of the symbols causes an overlap of the word onto itself: *y*_*i*_ = *y*_*i*+*t*_ for 1 ≤ *i* < *i* + *t* ≤ *d*. For example, the word *acacacac* is periodic with period 2, the word *acgtacgt* is periodic with period 4, while the word *acgaacgt* is not periodic.

Given a sequence *w* ∈ *A*^*n*^, with *w* = *x*_1_ *x*_2_, …*x*_*n*_, where *n* ≫ *d*, the probability of finding in it an occurrence of a periodic word *u*, that follows another occurrence of *u*, is relatively high. So, the independence condition of subsequent occurrences, needed to use the Poisson distribution, ceases to apply. Therefore it is necessary to introduce specific tricks, depending on the period of the string, in order to approximate the independence of occurrences.

Let us define the event *E*(*u*; *k*) as the case in which an instance of *u* occurs in *w* at position *k*. If *u* is not periodic and the sequence is stationary then the expected number of occurrences of *u* in *w* (i.e., the frequency of *u*), is

$$\begin{array}{c} \nu \left(u\right)=\sum_{k=1}^{n-d+1}P\left(E\left(u;k\right)\right)=P\left(E\left(u\right)\right)\left(n-d+1\right)\;, \end{array}$$ (9)

where *P*(*E*(*u*; *k*)) is the probability of *E*(*u*; *k*) to occur, that, using the stationarity of the sequence, can be written as *P*(*E*(*u*)). The case of periodic words is slightly more complicated. For periodic words *u* of period 1, i.e., sequences composed of just one symbol (e.g., *u* = *aaa*⋯*a*), it is sufficient to consider a generalized event *E*′(*u*; *k*) as *E*(*u*; *k*) coupled with event $x_{k+d}=\bar{a}$, where the event $\bar{a}$ indicates not *a*, so that if *E*′(*u*; *k*) occurs then *P*(*E*′(*u*; *k* + 1)) = 0, excluding subsequent occurrences of *u*. Summing up the case of periodic sequences of period 1 one has

$$\begin{array}{ccc} \nu (u) & = & \sum_{k=1}^{n-d}P\left(E^{\prime }\left(u;k\right)\right)+P(E\left(u;n-d+1\right)\\ & = & P\left(E^{\prime }\left(u\right)\right)\left(n-d\right)+P\left(E\left(u\right)\right)\;. \end{array}$$ (10)

The last term *P*(*E*(*u*)) accounts for the case the word *u* is at the end of the sequence, for which the event $x_{k+d}=\bar{a}$ is not defined. We can use the same strategy for the case of words with longer periods by introducing different generalized events, which guarantee independence of subsequent occurrences.

The probability *P*_0_(*u*), that the word *u* does not occur in the sequence *w*, can be approximated using the Poisson distribution $P\left(\#u=k\right)=\frac{\nu {\left(u\right)}^{k}}{k!}e^{-\nu (u)}$, which gives the probabilities of finding *k* occurrences of the word *u* in the sequence *w*, computed for *k* = 0:

$$\begin{array}{c} P_{0}\left(u\right)≃P\left(\#u=0\right)=e^{-\nu (u)}\;. \end{array}$$ (11)

The expected number of absent words in the sequence *w* is given by

$$\begin{array}{c} \left\langle \#H_{0}\right\rangle =\sum_{u\in U}P_{0}\left(u\right)=\sum_{u\in U}e^{-\nu (u)}\;. \end{array}$$ (12)

It is important to consider *P*_0_(*u*) for each different *u* because probabilities for different words could be several orders of magnitude different.

The probability of a word *u* of being a first order nullomer is obtained from the probabilities to be a nullomer of all the words obtained by mutating one symbol of *u*, i.e., all the word *v* ∈ *N*_1_(*u*). Each of the words *v* ∈ *N*_1_(*u*) can be a nullomer independently from the others, therefore one has

$$\begin{array}{c} P_{1}\left(u\right)=\prod_{v\in N_{1}\left(u\right)}P_{0}\left(u\right)≃\prod_{v\in N_{1}\left(u\right)}e^{-\nu (u)}=\exp(-\sum_{v\in N_{1}\left(u\right)}\nu \left(u\right))\;. \end{array}$$ (13)

Then, the expected number of nullomers of order 1 in the sequence *w* is

$$\begin{array}{c} \left\langle \#H_{1}\right\rangle =\sum_{u\in U}P_{1}\left(u\right)=\sum_{u\in U}\exp(-\sum_{v\in N_{1}\left(u\right)}\nu \left(u\right))\;. \end{array}$$ (14)

The generalization to k-th order nullomers is straightforward, considering the probabilities of being a nullomers of all the words in the set *N*_*k*_(*u*) of k-th order mutation of *u*.

It is worth noting that the above argumentation is general and does not depend on the specific random process underlying the generation of the symbolic sequence, since we have not specified *P*(*E*(*k*)). We conclude by giving the probabilities for the event *E*(*u*, *k*) in the Eqs (9) or (10) in the case of two different random processes, the first when each symbol has been extracted independently from the previous ones (independent nucleotides sequences), and the second when each symbol has a probability of occurring that depends on the previous symbol: in the first case

$$P\left(E\left(u;k\right)\right)=\prod_{j=1}^{d}p\left(x_{k+j-1}=y_{j}\right)=\prod_{j=1}^{d}p\left(y_{j}\right)=P\left(E\left(u\right)\right)\;,$$

in the second case

$$\begin{array}{ccc} P(E(u;k)) & = & \prod_{j=1}^{d}p\left(x_{k+j-1}=y_{j}|x_{k+j-2}=y_{j-1}\right)\\ & = & p\left(y_{1}\right)\prod_{j=2}^{d}W_{y_{j-1},y_{j}}=P\left(E\left(u\right)\right)\;, \end{array}$$ (15)

where *p*(*a*) is the probability of occurrence of the symbol *a* and *W*_*a*,*b*_ is the transition probability, that is the probability that a symbol *b* occurs after a symbol *a*.

### Genomes

Nullomers and high order nullomers were computed based on the genome sequences available from the NCBI database (https://www.ncbi.nlm.nih.gov/genome/). The analysis were performed focusing on the human genome (Homo sapiens build 38—GCF 000001405.35), but several other species were also included in this study: Bovine (Bos taurus—GCF 000003055.6), Chimpanzee (Pan troglodytes—GCF 000001515.7), Chicken (Gallus gallus—GCF 000002315.4), Goat (Capra hircus—GCF 001704415.1), Gorilla (Gorilla gorilla—GCF 000151905.1), Lemur (Microcebus murinus—GCF 000165445.1), Mouse (Mus musculus—GCF 000001635.25), Opossum (Monodelphis domestica—GCF 000002295.2), Rabbit (Oryctolagus cuniculus—GCF 000003625.3), Rat (Rattus norvegicus – GCF 000001895.5).

## Results

### Nullomers and high order nullomers in human genome

The first issue to be addressed concerns the number of nullomers and high order nullomers found in the genome: is it or is it not comparable with the expected number of nullomers and high order nullomers of a random sequence? As showed in [6] the number of nullomers in the human genome is much higher than the one of a random sequence, when considering a word of the same length and nucleotide frequencies. We extended the computation of human genome nullomers to high order nullomers, and we also computed nullomers and high order nullomers of random sequences preserving nucleotide and dinucleotide frequencies. Before presenting our results, it is worth remarking that nullomers of length *d*, larger than the minimum length at which nullomers appear for the first time, carry redundant information, since there are many of them that are trivially nullomers of previous lengths plus any symbol at the beginning or at the end of the sequence. Therefore, for our analysis, we always chose nullomers and high order nullomers at the minimum length (shortest high order nullomers). For example in the human genome, nullomers appear at length 11, while first and second order nullomers appear at length 14 and 16, respectively. We note that shortest simple nullomers and minimal absent words [7] (i.e., absent words that are not merely an extension of smaller nullomers) of corresponding size, coincide.

In Table 1 we report the number of nullomers of the human genome, #*H*_0_, and first order nullomers, #*H*_1_, for a size ranging from 11 to 14 nucleotides. The corresponding expected number of nullomers (obtained by Formulas (12) and (14)), namely 〈#*H*_0_〉_*nu*_, 〈#*H*_1_〉_*nu*_, 〈#*H*_0_〉_*di*_ and 〈#*H*_1_〉_*di*_, refer to random sequences of the same length of the human genome either with the same nucleotide frequencies (*nu*) or with the same dinucleotide frequencies (*di*). As expected, the rough hypothesis of random and independent nucleotides leads to value of 〈#*H*_0_〉_*nu*_ and 〈#*H*_1_〉_*nu*_ that are several orders of magnitude smaller than the corresponding numbers of real nullomers. On the contrary, the expected numbers of nullomers 〈#*H*_0_〉_*di*_ and 〈#*H*_1_〉_*di*_, in the case of random sequences preserving dinucleotide frequencies, are much higher than the numbers of nullomers in the human genome (see Table 1).

**Table 1:** For size ranging from 11 to 14—first column, we report: the number of occurring k-mers in human genome—second column; the number of nullomers in human genome, #*H*_0_—third column (the sum of the first two columns giving the total number of possible k-mers, i.e., 4^*d*^); the expected number of nullomers in the case of a random sequence of the same nucleotide frequencies, 〈#*H*_0_〉_*nu*_—fourth column; the expected number of nullomers in the case of a random sequence of the same dinucleotide frequencies, 〈#*H*_0_〉_*di*_—fifth column. The columns from 6 to 8 report first order nullomers for human genome, #*H*_1_ and the expected number of nullomers 〈#*H*_1_〉_*nu*_, 〈#*H*_1_〉_*di*_ in the case of random sequences preserving nucleotide and dinucleotide frequencies, respectively. The length of the random sequence is the same as the human genome (approximately 3.05 ⋅ 10^9^ base pairs). Simple and first order shortest nullomers are reported in bold.

d	Present	#*H*_0_	〈#*H*_0_〉_*nu*_	〈#*H*_0_〉_*di*_	#*H*_1_	〈#*H*_1_〉_*nu*_	〈#*H*_1_〉_*di*_
11	4194200	**104**	**6.08e-57**	**855.54**	0	0	1.116e-12
12	16732931	44285	3.42e-06	59922.2	0	0	0.286659
13	64761232	2347632	14.2219	1.53e+06	0	2.14e-150	412.653
14	227637490	40797966	248262	2.61e+07	**7874**	**1.07e-029**	**53798.5**

In order to shed light on this puzzling scenario we report the nucleotide and dinucleotide frequencies of the human genome:

$$\begin{array}{c} f_{nu}=(\begin{array}{c} 0.295\\ 0.205\\ 0.205\\ 0.295 \end{array})\;\;\;f_{di}=(\begin{array}{cccc} 0.098 & 0.050 & 0.070 & 0.077\\ 0.073 & 0.052 & 0.010 & 0.070\\ 0.060 & 0.043 & 0.052 & 0.050\\ 0.064 & 0.060 & 0.073 & 0.098 \end{array})\;, \end{array}$$ (16)

where rows and columns refer to nucleotide, *A*, *C*, *G* and *T* in this order. Those values clearly highlight a large non uniformity in the distribution of dinucleotides with respect to the nucleotide distribution. For example, according to the independent nucleotide hypothesis, all the couples *CC*, *CG*, *GC* and *GG* must appear with a probability of ∼0.042, but the actual value is ∼0.052 for *CC* and *GG*, ∼0.01 for *CG* and, finally ∼0.043 for *GC*. Moreover when computing the probability of a word to be a nullomer (according to Eq (11) or using Eq (13) for first order nullomer) the differences appear to be astonishing. For example the probability of being a nullomer for *CGCGCGCGCGC* is ∼7.9 ⋅ 10^−69^ according to the independent nucleotide hypothesis, while it is ∼8.6 ⋅ 10^−1^ (68 orders of magnitude higher) when preserving dinucleotide frequencies (being the difference in word frequency, *ν*, deeply amplified by the exponentiation in Eq (11)).

The above example reveals how CpG content, that is the dinucleotide with the lowest frequency, deeply influences the probability of a sequence to be a nullomer. This analysis seems to confirm the hypothesis of [5] claiming that nullomers are trivially the consequence of the hypermutability of CpG dinucleotides. Anyway, further investigations on CpG composition of nullomers show a more complex scenario.

**Fig 1:**
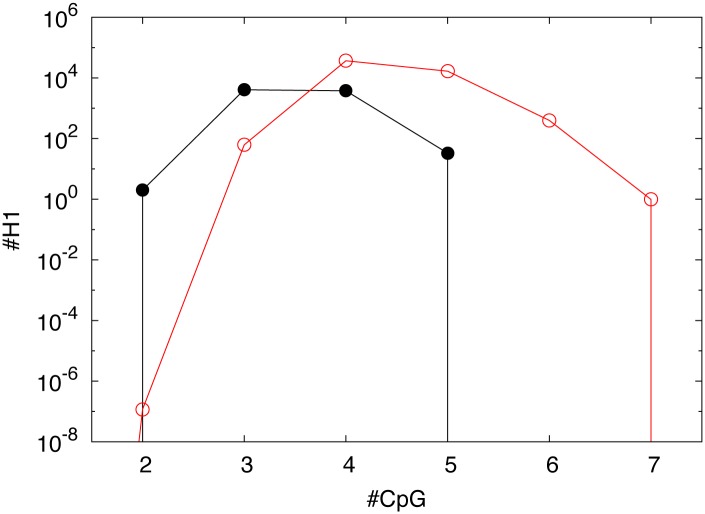
Number of first order nullomers (black filled circles, ⚫) compared with expected number of first order nullomers (red empty circle, ⚪) of size 14, as a function of the number of CpGs occurring in the sequences. The expected number of nullomers is computed considering random sequences with the same length of the human genome preserving dinucleotide frequencies.

In Fig 1 we compare the number of first order nullomers of length 14 of the human genome with that of random sequences preserving dinucleotide frequencies, for each possible number of dinucleotide CpGs (from 0 to 7) occurring in the sequence. The figure highlights that the total number of nullomers is smaller than expected, as previously stated (see Table 1). More importantly, Fig 1 shows that real nullomers are distributed according to the CpG content differently than expected by chance.

This is particularly evident for CpG content 6 (CpG content 7 being trivially made of one sequence) where no real nullomer was found, even though one would expect a very high number of nullomers. Remarkably for CpG content 2 the opposite scenario is shown, where the expected number is much lower than the real number. The above consideration states that, even if CpG content strongly affects the probability of being a nullomer, it is not the only ingredient in determining human nullomers (see also [9]). Such a scenario can be explained by the presence in genomes of large fragments with a high frequency of CpG dinucleotide, known as CpG islands. Those regions cause an over representation of k-mers with a high CpG content leading to an under representation of them as nullomers. In S1 Appendix (Supporting Information) we introduce a model to generate random sequences with artificial CpG islands, preserving dinucleotide frequencies. By tuning the rate of CpG aggregation of the model we easily obtained random sequences with the same number of nullomers of genuine genome sequences. Anyway the structural features of the obtained nullomers are still very different from real ones as shown in the S1 Appendix. Therefore, even if the CpG aggregation has a deep impact on nullomers, the non trivial structure of the real nullomers cannot be obtained from a model that randomly clusters CpG dinucleotides.

### CpG frequencies along nullomers

As reported in [15] it has been observed that motifs containing CpGs are underrepresented in vertebrates due to the hypermutability of CpGs [8], so that oligomers containing CpGs tend to occur less and less than other sequences. Hypermutability of CpG is for sure one of the most important force driving nullomer phenomenon, as confirmed by the CpG abundance in simple and high order nullomers, even if, as shown in the previous section, hypermutability model is not sufficient to completely explain the features of nullomers and high order nullomers. In this section we confirm this statement by analyzing the distribution of CpG along nullomers sequences, computing the percentage of CpG dinucleotide for each position of the sequence.

**Fig 2:**
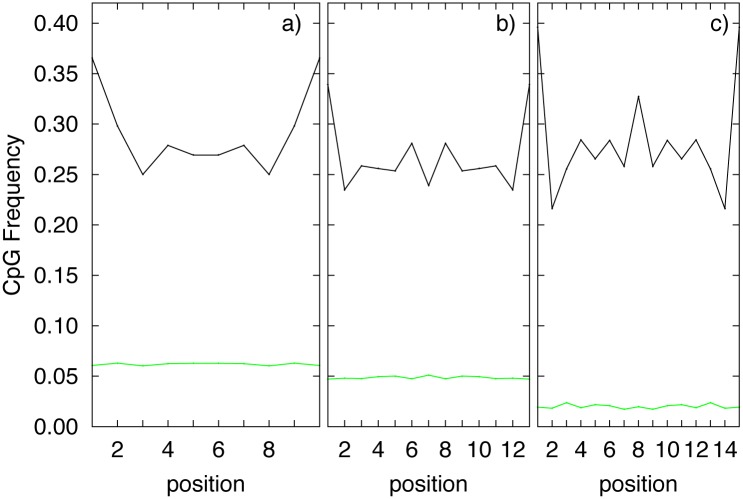
CpG frequencies for each dinucleotide position (black line) for $H_{0}^{11}$ (panel a), $H_{1}^{14}$ (panel b) and $H_{2}^{16}$ (panel c) of the human genome. CpG frequencies for present sequences of the same length (green line) are also reported in the three panels.

In Fig 2 black plots show the frequencies of CpG dinucleotide in nullomers for $H_{0}^{11}$ (panel a), $H_{1}^{14}$ (panel b), $H_{2}^{16}$ (panel c), and for present sequences of the same lengths (for comparison purposes, green plots). The three black plots in panel a—b and c clearly show a strong dependence of the frequencies on the position in the sequence, with respect to a quite uniform profile in the case of present sequences. The strongest dependence can be observed in panel c, where the percentage of CpG content ranges from around 23% till around 37%. It is worth noting that the difference in average CpG frequency for the present sequences (green plots) showed in the three panels of Fig 2, depends on the different ratio between absent, with a high CpG content, and present sequences. Moreover all the plots are symmetric because the complementary reverse sequence of a nullomer is still a nullomer.

**Fig 3:**
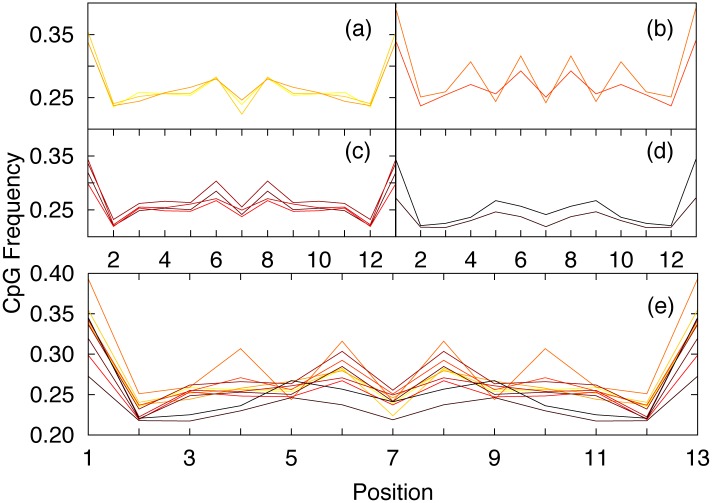
CpG frequencies for each dinucleotide position for first order nullomers ($H_{1}^{14}$) in eleven different species: panel *a*) Human, Chimpanzee and Gorilla (yellow, dark yellow and light orange, respectively), panel *b*) Rat and Mouse (orange and light red, respectively), panel *c*) Opossum, Bovine, Goat and Lemur (red, dark red, light brown and brown, respectively), panel *d*) Chicken, and Rabbit (very dark brown and black, respectively). In panel *e* all the species are reported together.

The analysis of CpG dinucleotide frequencies was extended to other species in order to confirm the presence of peculiar patterns serving as a fingerprint of specific species. In Fig 3 (panel e) CpG frequencies are reported for $H_{1}^{14}$ for all the eleven species considered in this work. Again the patterns are clearly non uniform, and also in this case there are very high frequencies for the first and last dinucleotides. Moreover the 11 species were divided into four groups according to their CpG frequency profiles (panel a, b, c and d). It is worth noting how close species typically show similar patterns. This is particularly evident in panel a) where Human, Gorilla and Chimpanzee are grouped together sharing very similar CpG patterns. However, the other panels (panel b) Rat and Mouse, panel c) Opossum, Bovine, Goat and Lemur and panel d) Chicken, and Rabbit) also show very similar and peculiar patterns, indicating that nullomers related to close species present very similar CpG frequency patterns.

### Phylogenetic trees based on simple and high order nullomers

In order to quantify to what extent nullomers have been conserved among close species during evolution, we designed two different similarity functions. Let us consider nullomers of order *s* for sequence size *d* of two genomes named *g*_*h*_ and *g*_*k*_. The first function, $DJ_{s}^{d}\left(g_{h},g_{k}\right)$, based on the Jensen-Shannon entropy [16], measures the similarity between the dinucleotide distributions for each position of nullomer sequences of two genomes, and it is defined as follows

$$\begin{array}{ccc} & & DJ_{s}^{d}\left(g_{h},g_{k}\right)=\\ & & \;\sum_{j=1}^{d-1}\sum_{i\in DI}(-\frac{p_{h}^{j}\left(i\right)+p_{k}^{j}\left(i\right)}{2}\;\log(\frac{p_{h}^{j}\left(i\right)+p_{k}^{j}\left(i\right)}{2})+\\ & & \;\frac{p_{h}^{j}\left(i\right)\;\log\left(p_{h}^{j}\left(i\right)\right)}{2}+\frac{p_{k}^{j}\left(i\right)\;\log\left(p_{k}^{j}\left(i\right)\right)}{2}) \end{array}$$ (17)

where $p_{z}^{j}\left(i\right)$ is the frequency of dinucleotide *i* at position *j* for nullomers of genome *g*_*z*_ for *z* = *h*, *k*. For each couple of genomes the distance $DJ_{s}^{d}\left(g_{h},g_{k}\right)$ is computed as the sum of Jensen-Shannon entropies of the dinucleotide distributions for each dinucleotide position.

The second distance, $DC_{s}^{d}\left(g_{h},g_{k}\right)$, based on the number of nullomer sequences two genomes share, is defined as follows

$$\begin{array}{c} DC_{s}^{d}\left(g_{h},g_{k}\right)=1-\frac{∥\;H_{n}^{d}\left(g_{h}\right)\cap H_{n}^{d}\left(g_{k}\right)\;∥}{\min_{p=h,k}\;(∥H_{n}^{d}\left(g_{p}\right)∥)} \end{array}$$ (18)

For each couple of genomes the distance $DC_{s}^{d}\left(g_{h},g_{k}\right)$ is computed as the number of *s*-th order nullomers they share, divided by the size of the smallest nullomer set.

Eleven different organisms were considered and two sets of nullomers $H_{0}^{11}$ and $H_{1}^{14}$ were used. We obtained four distances $DC_{0}^{11}\left(g_{h},g_{k}\right)$, $DC_{1}^{14}\left(g_{h},g_{k}\right)$, $DJ_{0}^{11}\left(g_{h},g_{k}\right)$ and $DJ_{1}^{14}\left(g_{h},g_{k}\right)$ that were used to assess phylogenetic relationships among species. UPGMA algorithm [17], implemented in Phylip package (http://cmgm.stanford.edu/phylip/), was used to generate four phylogenetic trees: T1 related to $DC_{0}^{11}\left(g_{h},g_{k}\right)$, T2 related to $DC_{1}^{14}\left(g_{h},g_{k}\right)$, T3 related to $DJ_{0}^{11}\left(g_{h},g_{k}\right)$ and T4 related to $DJ_{1}^{14}\left(g_{h},g_{k}\right)$.

**Fig 4:**
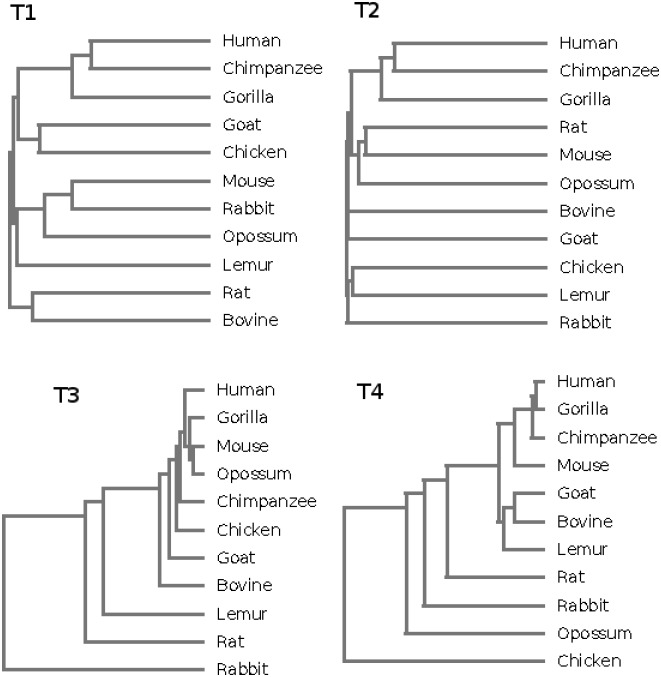
Phylogenetic trees of 11 species obtained by (first row) DC distance for nullomers (T1—on the left) and first order nullomers (T2—on the right); (second row) DJ distance for nullomers (T3—on the left) and first order nullomers (T4—on the right).

All the obtained phylogenetic trees in Fig 4 show a reasonable level of accuracy with respect to classical phylogeny. The three species belonging to Primates (Homo sapiens, Pan trogloditis and Gorilla gorilla) are placed in the same branch of the trees except T3, with human and chimpanzee closer than gorilla in T1 and T2. Rattus norvegicus and Mus musculus are placed in the same branch in T2, likewise Goat and Bovine in T2 and T4. T4 places Chicken apart from all the other organisms. The trees T2 and T4, based on first order nullomers of size 14, show an overall higher accuracy with respect to T1 and T3, based on simple nullomers of size 11, indicating that higher order nullomers seem to be more conserved among close species.

Although the phylogenetic trees show some incongruities, close related species tend to share large portions of their nullomers or tend to show similar sequences in terms of dinucleotide composition of nullomers. Therefore, albeit phylogeny is out of scope for this paper, those phylogenetic trees confirm the non-random nature of nullomers (see also [9]).

### Helical rise of nullomers

The hypothesis, supported by the analysis of CpG frequencies, that nullomers are characterized by peculiar structural features, pushed us to investigate the chemico-physical properties of those oligomers. Could they have potentially negative effects on DNA three dimensional organization and stability? According to [18] the lengths of dinucleotide steps of DNA helix, known as helical rise, can influence the propensity of oligomers to bind the histone complex to form stable nucleosomes. In particular high values of DNA helical rise can ease the formation of a strong nucleosome and short sequences with a significantly high mean helical rise can bind the histone octamer core.

The helical rise tetranucleotide code for the 136 possible tetrads was used according to [19]. Each value of the code refers to the central dinucleotide helical step, taking into account the first two flanking bases. For instance, a value of 3.40Å is assigned to ACTG tetrad, meaning that the central dinucleotide CT has such a value if it occurs with an A and a G as flanking bases. The mean value of the helical rise distribution is 3.2Å with a maximal and a minimal value of 4.46Å (CGCA/TGCG) and 2.36Å (ATGA/TCAT), respectively, with a remarkable difference of 2.1Å between these two values.

**Fig 5:**
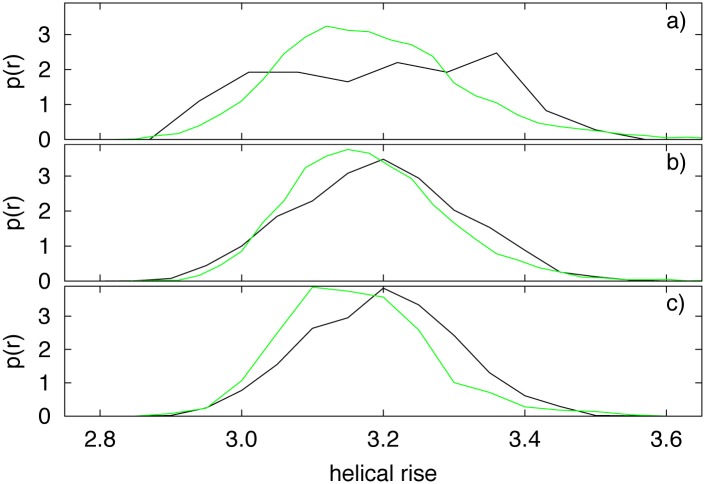
Distribution of average rise values (black line) for $H_{0}^{11}$ (panel a), $H_{1}^{14}$ (panel b) and $H_{2}^{16}$ (panel c). Average rise values for present sequences (green plot) are also reported in the three panels.

Using the tetranucleotide code the helical rise profiles of nullomers were computed and analyzed. In Fig 5 the distributions of mean helical rise of $H_{0}^{11}$, $H_{1}^{14}$, $H_{2}^{16}$ and corresponding present sequences are reported. As one can observe the mean helical rise is significantly higher in nullomers than in present sequences. This is particularly evident in panel c) for $H_{2}^{16}$ but also in panel a) and b) the average rise value distributions appear to be significantly different with a propensity of nullomers sequences to have higher mean rise values. These results support our hypothesis that nullomers are the product of a CpG hypermutability process as well as the consequence of the complex structure of genomes.

## Discussion

The set of nullomers of a given DNA sequence is composed by all the oligomers that do not occur as substrings in that DNA sequence. In this paper we investigated the nature of nullomers, trying to address the question: is it just a statistical matter or is it the consequence of the peculiar features of genomic sequences? In this context we proposed an extension of the notion of nullomer introducing high order nullomers, i.e. nullomers such that each of their mutated sequences is still a nullomer. High order nullomers could emphasize the features that already make simple nullomers useful in several applications, as confirmed by the analysis on phylogenetic trees. We compared nullomers and high order nullomers of the human genome with those expected for a random sequence of the same length, generated with different stochastic processes, i.e. using the same nucleotide or dinucleotide frequencies. Moreover we implemented a model to generate random sequences with artificial CpG islands, preserving dinucleotide frequencies (see Supporting Information). We obtained that, in any case, real nullomers have very different statistical properties from those obtained from random sequences, leading to the conclusion that the CpG hypermutability model (as proposed in [5]), also taking into account CpG islands, is not sufficient in explaining the nature of nullomers (as already asserted in [9]). In particular we showed that CpG content and CpG frequencies (being the frequencies considered as a function of dinucleotide position) of nullomers in random sequences with both the same dinucleotide frequencies and the same number of nullomers of a given genome, are substantially different from those of real nullomers.

Furthermore, in real genomes, the CpG dinucleotide frequencies computed as a function of dinucleotide position, cluster into homogeneous groups according to their CpG frequency profiles and close species typically show similar patterns. We built phylogenetic trees using a distance based on either the dinucleotide frequencies or the number of nullomers that two different species share, demonstrating in both cases that nullomers are well conserved among close species.

In order to provide insights on the origin of nullomers, we investigated whether those sequences have some peculiar structural feature with potential harmful effects on DNA. We found that the mean helical rise values for nullomers sequences are essentially higher than the ones of present sequences, suggesting, according to [18], a potential strong interaction with histone core complex. This could be the reason that led them to be removed; this is just an hypothesis that should be confirmed by experimental evidence but that is supported by our in silico analysis.

Recently, nullomers have attracted some interest in the literature because of their possible relevance in different fields, from the identification of pathogen-specific signature [20] to drug discovery [21]. In [20] the authors identified short DNA sequences of Ebola virus that are simple nullomers for human. Those sequences could be used as pathogen-specific signatures for quick and precise action against infectious agents. Furthermore, the studies on nullomers in proteomes led to interesting works, based on the assumption that absent small peptides could be harmful for the cell, similarly to the hypothesis we propose concerning genomic nullomers. Some of those peptides were tested on normal and cancer cells showing different lethal effects [21]. In those contexts, it could be of interest to test the efficacy of high order nullomers.

Moreover it has been shown how nullomers can have a practical relevance in forensic genomics [22], since they can be used for tagging casework samples, as a sort of nullomer barcode, being that tagging nullomers are naturally absent. The introduction of high order nullomers could be extremely useful in this context, since even if mutations occur, high order nullomers guarantee the sequence to still be absent.

A final consideration is devoted to CpG islands and hypermutability model and the role they play in the biological basis of the nullomer phenomenon. In this work we compare nullomers (and their statistical properties) of real genome with those obtained from random sequences generated with the same statistical properties (dinucleotide frequencies and CpG islands) of real genome. A very challenging problem is the design of a biologically based hypermutability model able to derive the properties of CpG islands and their relationships with the nullomer phenomenon. A detailed and comprehensive investigation on this topic could be the subject of a new study.

## Supporting Information

S1 Appendix A model to generate CpG-clustered random sequences. (PDF)

## References

[1] Fleischmann RD, Adams MD, White O, Clayton RA, Kirkness EF, Kerlavage AR, Bult CJ, Tomb JF, Dougherty BA, Merrick JM. Whole-genome random sequencing and assembly of Haemophilus influenzae rd. Science. 1995;269:496–512 10.1126/science.7542800 7542800

[2] Karlin S, Mrazek J, Campbell AM. Compositional biases of bacterial genomes and evolutionary implications. Journal of bacteriology. 1997;179:3899–3913 10.1128/jb.179.12.3899-3913.1997 9190805 PMC179198

[3] Karlin S, Mrazek J. Compositional differences within and between eukaryotic genomes. Proceedings of the National Academy of Sciences. 1997;94:10227–10232 10.1073/pnas.94.19.10227 PMC23344 9294192

[4] Hampikian G, Andersen T. Absent sequences: nullomers and primes. Pacific Symposium on Biocomputing. 2007;12:355–366. 17990505 10.1142/9789812772435_0034

[5] Acquisti C, Poste G, Curtiss D, Kumar S. Nullomers: really a matter of natural selection? PloS one. 2007;2:1022 10.1371/journal.pone.0001022 17925870 PMC1995752

[6] Herold J, Kurtz S, Giegerich R. Efficient computation of absent words in genomic sequences. BMC bioinformatics. 2008;9:167–175 10.1186/1471-2105-9-167 18366790 PMC2375138

[7] Pinho AJ, Ferreira PJ, Garcia SP, Rodrigues JM. On finding minimal absent words. BMC bioinformatics. 2009;10:137–147 10.1186/1471-2105-10-137 19426495 PMC2698904

[8] Sved J, Bird A. The expected equilibrium of the cpg dinucleotide in vertebrate genomes under a mutation model. Proceedings of the National Academy of Sciences. 1990;87(12):4692–4696 10.1073/pnas.87.12.4692 2352943 PMC54183

[9] Garcia SP, Pinho AJ, Rodrigues JM, Bastos CA, Ferreira PJ. Minimal absent words in prokaryotic and eukaryotic genomes. PLoS ONE. 2011;6(1):16065 10.1371/journal.pone.0016065 PMC3031530 21386877

[10] Chairungsee S, Crochemore M. Using minimal absent words to build phylogeny. Theoretical Computer Science. 2012;450:109–116 10.1016/j.tcs.2012.04.031

[11] Goswami J, Davis MC, Andersen T, Alileche A, Hampikian G. Safeguarding forensic DNA reference samples with nullomer barcodes. Journal of forensic and legal medicine. 2013;20(5):513–519 10.1016/j.jflm.2013.02.003 23756524

[12] Guibas LJ, Odlyzko AM. String overlaps, pattern matching, and nontransitive games. Journal of Combinatorial Theory. 1981;30(2): 183–208 10.1016/0097-3165(81)90005-4

[13] Rahmann S, Rivals E. Exact and efficient computation of the expected number of missing and common words in random texts In: Combinatorial Pattern Matching. Springer 2000:375–387 10.1007/3-540-45123-4_31

[14] Rahmann S, Rivals E. On the distribution of the number of missing words in random texts. Combinatorics, Probability and Computing. 2003;12(01):73–87 10.1017/S0963548302005473

[15] Josse J, Kaiser A, Kornberg A. Enzymatic synthesis of deoxyribonucleic acid. J biol chem. 1961;236(3):864–875 13790780

[16] Lin J: Divergence measures based on the shannon entropy. IEEE Transactions on Information Theory. 1991;37(1):145–151 10.1109/18.61115

[17] Sokal RR. A statistical method for evaluating systematic relationships. Univ Kans Sci Bull. 1958;38:1409–1438

[18] Pedone F, Santoni D. Preferential nucleosome occupancy at high values of DNA helical rise. DNA research. 2012:043 10.1093/dnares/dsr043 PMC3276262 22233711

[19] Pedone F, Santoni D. Sequence-dependent DNA helical rise and nucleosome stability. BMC molecular biology. 2009;10(1):105 10.1186/1471-2199-10-105 19943916 PMC2788551

[20] Silva RM, Pratas D, Castro L, Pinho AJ, Ferreira PJ. Three minimal sequences found in Ebola virus genomes and absent from human DNA. Bioinformatics. 2015; 31:2421–2425. 10.1093/bioinformatics/btv189 25840045 PMC4514932

[21] Alileche A, Goswami J, Bourland W, Davis M, Hampikian G. Nullomer derived anticancer peptides (nullops): Differential lethal effects on normal and cancer cells in vitro. Peptides. 2012;38(2):302–311 10.1016/j.peptides.2012.09.015 23000474

[22] Goswami J, Davis MC, Andersen T, Alileche A, Hampikian G. Safeguarding forensic DNA reference samples with nullomer barcodes. Journal of forensic and legal medicine. 2013;20(5):513–519 (2013) 10.1016/j.jflm.2013.02.003 23756524

