# Supplementary material for: Nullomers and High Order Nullomers in Genomic Sequences
Source: PLoS One. 2016 Dec 1;11(12):e0164540. doi: 10.1371/journal.pone.0164540 (PMC5132333; doi:10.1371/journal.pone.0164540)
Supplement: S1 Appendix — (PDF) [file pone.0164540.s001.pdf]

Supporting Information

S1 Appendix

In this appendix we present a simple algorithm able to modify a given genomic sequence, preserving dinucleotide frequencies, in order to generate artificial CpG islands, as observed in DNA human genome. In this way we can compare nullomers of a real genomic sequence with those of a random sequence with the same dinucleotide frequencies and a similar clustering structure of CpG dinucleotides.

Starting from a random sequence with a uniform distribution of nucleotides along the sequence, we generate artificial CpG islands by clustering CpG dinucleotides. We use a stochastic rule that swaps with a given probability a CpG dinucleotide with a non CpG dinucleotide when the flanking bases of the two dinucleotides are the same, so that dinucleotide frequencies are conserved. The probability to swap two such dinucleotides depends on the density of CpG around the two dinucleotides in such a way that it is likely to move CpG dinucleotides from regions with a low CpG density to regions with a high CpG density. As a consequence the distribution of CpG dinucleotides along the sequence is no more uniform and regions with a high density of CpG dinucleotides appear, mimicking the presence of CpG islands.

Given a sequence, $w \in A^n = x_1 x_2 ... x_n$, as first we define the function $H(i;\ell)$ as the number of CpG dinucleotides around the position i in a window of length 2ℓ, i.e. in the interval $[i-\ell : i+\ell]$, letting $i-\ell = 1$ if $i-\ell < 1$ and $i+\ell = n$ if $i+\ell > n$. We define the swap step in the following way:

- we pick at random a CpG dinucleotide, say at position i;
- we pick at random a non CpG dinucleotide, say at position $j \neq i$ such that $x_{i-1} = x_{j-1}$ and $x_{i+2} = x_{j+2}$, in order to maintain unchanged the dinucleotide frequencies;
- with probability $p = 1 - \min(H(i;\ell)/H(j;\ell), 1)$, we swap the dinucleotide CpG at position i with the dinucleotide $x_j x_{j+1}$ at position j.

In this way, regions with high CpG content tend to attract other CpGs and, as the number of swaps increases, also the clustering of CpG increases.

In order to compare random clustered sequence and real genomes we chose the first chromosome (chr1) of the human genome and a random sequence with the same length and dinucleotide frequencies. We chose only chr1, whose length is about 230Mb, for computational reasons, since the swapping procedure is largely memory and time consuming. The size of k-mers at which the first simple nullomers appear for chr1 is 10. We clustered the CpG in the random sequence making a given number of swaps, N_s, and we computed the number of nullomers of length 10 for the sequence at varying N_s.

Fig. 1 shows how initially the number of nullomers decreases at increasing the number of swaps, and it vanishes at approximately $N_s \approx 5 \cdot 10^5$. When the number of swaps approaches $N_s \approx 5.7 \cdot 10^6$, the number of nullomers starts to increase at increasing the number of swaps. This puzzling behavior can be explained as follows: when applying the CpG clustering algorithm to a sequence the aggregation degree of CpGs increases at

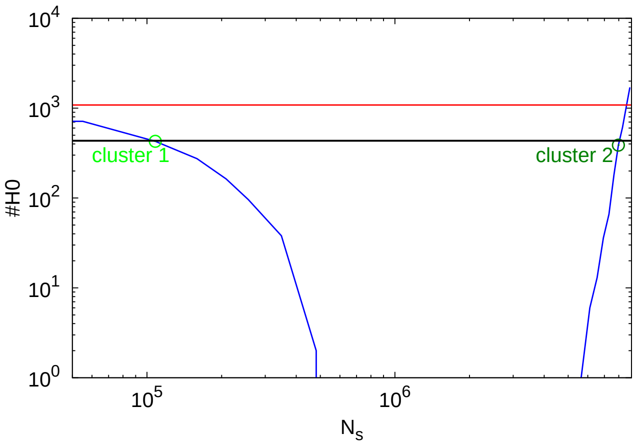

Figure 1: Number of simple nullomers of size 10, $\#H0$, as a function of the number of clustering swaps, N_s. The black solid line represents the number of nullomers for chr1 (i.e., 434 nullomers), and the red solid line represents the number of nullomers for the random sequence without clustering (i.e., 1083 nullomers). The circles (green – cluster 1 on the left and dark green – cluster 2 on the right), represents the chosen clustering level with the same (approximately) number of nullomers as in the real sequence.

increasing the number of swaps. We can observe that the number of nullomers clearly decreases till the number of swaps reaches $\approx 5 \cdot 10^5$. This is due to an over representation of k-mers with a high CpG content and a consequent under representation of those k-mers in the nullomers set, resulting in an overall drop of nullomers. When the number of swaps is higher than $\approx 5.7 \cdot 10^6$ the aggregation of CpGs is "too high" (a large number of CpG are aggregated) and the effect is the formation of multiple occurrences of sequences with a high CpG content, with a consequent under representation of k-mers with a low CpG content. The final result is that k-mers with a low CpG content will be absent i.e. nullomers, so that the number of nullomers grows up again.

In order to compare nullomers of chr1 to those obtained with random clustered sequences, we selected two clustered sequences with, approximately, the same number of nullomers of chr1, and we called them cluster 1 and cluster 2. As first it is useful to compare the structure of CpG clusters for chr1 and clusters 1 and 2. In Fig. 2 it is shown the number of occurrences, in the sequences, of words made of repeated CpG dinucleotides (e.g. "CGCGCGCG") as a function of the number of consecutive CpG. It is apparent that for clusters 1 and 2 the occurrences of consecutive CpG exponentially decrease at increasing the number of CpG in the word (as expected for random sequences) while for chr1 the behavior is not so trivial, showing a more complex structure (at least in terms of number of words containing only CpG dinucleotides) of chr1.

Finally, in Fig. 3 the comparison between the structural features of nullomers in terms of CpG content for chr1 and clusters 1 and 2 is shown. The graphs show that both the number of CpG occurring in nullomers (on the left) and the CpG frequencies in nullomers depending on the position (on the right) significantly differ between actual chr1 and random ones, either

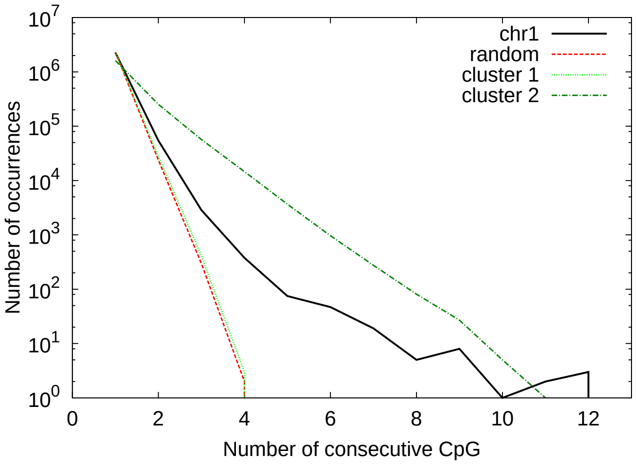

Figure 2: Number of occurrences of words containing only CpG dinucleotides, as a function of the number of CpG present in the word. The black solid line indicates chr1, the red dashed line the pure random sequence, the green dotted line the cluster 1 sequence and the dark green dashed dotted line the cluster 2 sequence.

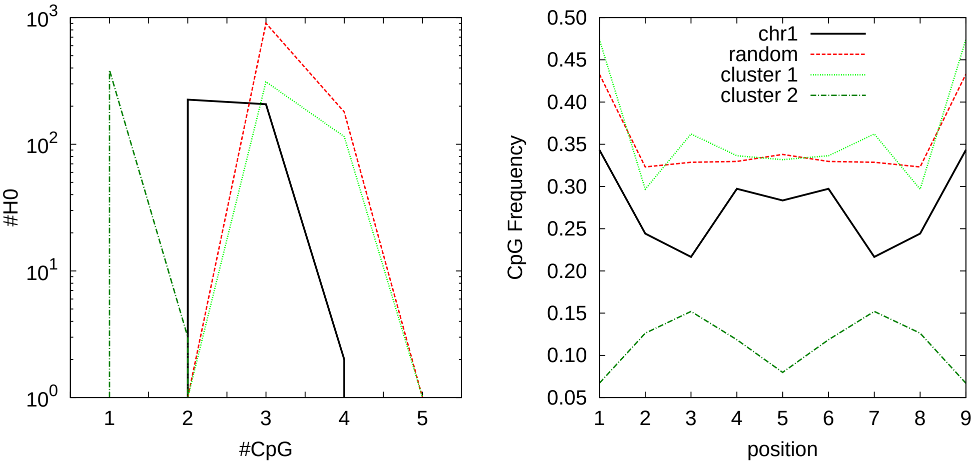

Figure 3: Left: number of simple nullomers of size 10 as a function of the number of CpGs occurring in the sequences. Right: CpG frequencies in nullomers for each dinucleotide position. The black solid line indicates chr1, the red dashed line the pure random sequence, the green dotted line the cluster 1 sequence and the dark green dashed dotted line the cluster 2 sequence.

clustered or not. This important result shows that the hypermutability model, also enriched with different kind of clustering of CpG dinucleotide, is not sufficient to reproduce the real structure of nullomers.
